# Credit to Whom Credit Is Due

**Published:** 1853-09

**Authors:** 


*Credit to whom Credit is due.*

WE have observed an article in some of our exchanges on *Stomatitis Materni*, taken from our June number, and credited to the *Med. Gazette.* We presume it is a mistake.

CORRECTION.—We notice a paragraph in some of our exchanges to the effect that the late Dr. G. W. Richards, of Dubuque, was at one time connected with Rush Medical College. This is a mistake; Dr. Richards was never, at any time, in any manner, connected with this institution.

